# The Roles of CircRNAs in Bladder Cancer: Biomarkers, Tumorigenesis Drivers, and Therapeutic Targets

**DOI:** 10.3389/fcell.2021.666863

**Published:** 2021-07-19

**Authors:** Fajuan Cheng, Bin Zheng, Shubin Si, Jianwei Wang, Guiting Zhao, Zhongshun Yao, Zhihong Niu, Wei He

**Affiliations:** ^1^Department of Nephrology, Shandong Provincial Hospital Affiliated to Shandong First Medical University, Jinan, China; ^2^Department of Nephrology, Shandong Provincial Hospital, Cheeloo College of Medicine, Shandong University, Jinan, China; ^3^Department of Urology, Shandong Provincial Hospital Affiliated to Shandong First Medical University, Jinan, China; ^4^Department of Urology, Shandong Provincial Hospital, Cheeloo College of Medicine, Shandong University, Jinan, China; ^5^Department of Urology, People’s Hospital of Yiyuan County, Zibo, China; ^6^Department of Urology, Shandong Provincial ENT Hospital Affiliated to Shandong University, Jinan, China

**Keywords:** circular RNA, bladder cancer, biomarker, diagnosis, prognosis, treatment

## Abstract

Bladder cancer (BCa) is the most prevalent malignancy of the urinary system. Circular RNAs (circRNAs), a novel subtype of non-coding RNAs, play a crucial role in physiological and developmental processes. CircRNAs mainly function as regulators of splicing process and transcription, microRNA sponges, and protein brackets. Recent advances in understanding the pathogenesis of BCa have led to the identification of an abundance of dysregulated circRNAs associated with BCa. These aberrantly expressed circRNAs eventually lead to abnormalities in biological, genetic, and epigenetic information. In this review, we introduce the potential of circRNAs as biomarkers for BCa diagnosis and prognosis. Notably, diverse mechanisms have been proposed for circRNAs driving carcinogenesis, including increasing cell proliferation, promoting invasive and migratory capacity, enhancing endothelial–mesenchymal transition, sustaining stemness, and enabling resistance to chemotherapy. Importantly, a full understanding of circRNA mechanisms is needed to mine promising therapeutic approaches for targeting BCa. In this paper, we present the latest advances in circRNAs and systemically summarize the characteristics and mechanisms of circRNAs in BCa, providing potential perspectives for BCa treatment.

## Introduction

Bladder cancer (BCa) is the most prevalent malignancy of the urinary system. Each year, BCa accounts for an estimated 500,000 new cases and 200,000 deaths worldwide, making it the fourth most frequent cancer in males and the 11th most frequent cancer in females (Richters et al., 2020; Siegel et al., 2020). Bladder urothelial carcinoma is the most common histologic subtype. Bladder urothelial carcinoma is a heterogeneous neoplasm originating from the urothelium, non-muscle-invasive bladder cancer (NMIBC), muscle-invasive bladder cancer (MIBC), or metastatic disease. The mainstay treatment for NMIBC is complete transurethral resection of the tumor followed by intravesical therapy. Multimodal treatment involving radical cystectomy with neoadjuvant systemic therapy is required for MIBC to control the disease or relieve symptoms. Systemic cisplatin-based cytotoxic chemotherapy or immunotherapy offers the best opportunity for the cure of advanced disease (Kamat et al., 2016). Advances in the understanding of BCa biology over the past decade have greatly expanded the treatment armamentarium (Patel et al., 2020). However, BCa is still associated with high morbidity and mortality rates (Witjes et al., 2021). Thus, there is an urgent need to further elucidate the genetic and molecular biology of BCa to explore more effective therapeutic targets.

Accumulating research has confirmed that genetic mutations and epigenetic alterations are involved in the carcinogenesis, development, and metastasis of BCa (Tran et al., 2020). Extensive studies have been performed on the tumorigenesis, progression, and therapeutic utilities of BCa, in which non-coding RNAs (ncRNAs), including microRNAs (miRNAs) (Deng et al., 2014; Yang et al., 2016; Liang et al., 2017), long ncRNAs (lncRNAs) (Chen C. et al., 2020; He X. et al., 2020; Zhan et al., 2020), and circle RNAs (circRNAs) (Bi et al., 2019; Lu et al., 2019; Su et al., 2020), are involved. Thus, targeting circRNAs is a promising area of potential clinical interest.

Circular RNAs, a novel subtype of ncRNAs, were first discovered as viroids in 1976 (Sanger et al., 1976) and were subsequently found in eukaryotes (Hsu and Coca-Prados, 1979; Kos et al., 1986; Matsumoto et al., 1990). CircRNAs are single-stranded, covalently closed loops that confer higher resistance to exonucleases (Patop et al., 2019). CircRNAs were initially considered functionless by-products resulting from erroneous pre-messenger RNA (pre-mRNA) splicing (Cocquerelle et al., 1993). However, recent advances and the application of high-throughput RNA sequencing (RNA-seq) technologies and bioinformatics have identified an abundance of diverse circRNAs that play a pivotal role in physiological and developmental processes (You et al., 2015; Szabo and Salzman, 2016; Du et al., 2017b; Aufiero et al., 2019). Moreover, increasing evidence has demonstrated the significance of circRNAs in tumorigenesis, proliferation, progression, and treatment resistance (Xiao et al., 2019, 1; Chen L. et al., 2020; Ye et al., 2020). Owing to their high stability, tissue specificity, and existence in exosomes and body fluids, circRNAs may be promising biomarkers or therapeutic targets for malignancies (Bahn et al., 2015; Rybak-Wolf et al., 2015; Li and Han, 2019). There has been increasing research to elucidate the characteristics and mechanisms of circRNAs in BCa to develop novel precision-medicine biomarkers and identify potential therapeutic targets. In this paper, we systematically summarize the characteristics, biogenesis, and pathogenesis of circRNAs in BCa and the potential prospects for targeting circRNAs for BCa treatment.

## Biogenesis and Characteristics of Circular RNAs

**FIGURE 1:**
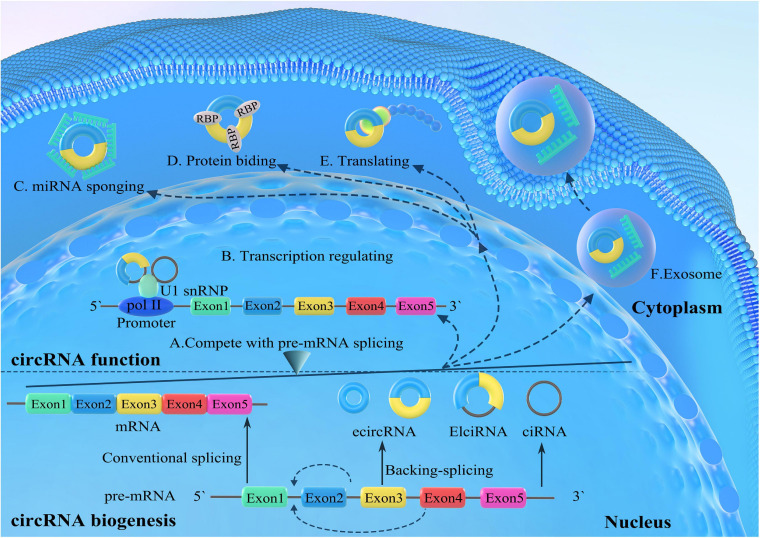
Biogenesis and functions of circular RNAs (circRNAs). The canonical splicing excludes the introns from immature pre-messenger RNA (pre-mRNA) to generate mature mRNAs. CircRNAs are derived from the unique back-splicing that covalently attaches the 3′ splice acceptor to the downstream 5′ splice donor, forming closed-loop structures, including exonic circRNAs (ecircRNAs), exon-intron circRNAs (eicircRNAs), and intronic circRNAs (ciRNAs). Generation of circRNAs competes with pre-mRNA splicing. CircRNAs regulate the transcription and splicing of parental genes by interacting with RNA polymerase II or transcription-related factors. CircRNAs competitively bind miRNAs and serve as intracellular competitive endogenous RNAs (ceRNAs), eliminating the suppressive effect of miRNAs on target genes. CircRNAs can also interact with RNA-binding proteins (RBPs) and serve as protein baits or antagonists, thus inhibiting the function of proteins. Some circRNAs are able to be translated into proteins. In addition, circRNAs can be transported into the extracellular space and adjacent cells or body fluids for regulation activities.

CircRNAs are synthesized during the posttranscriptional splicing of pre-mRNA, where canonical splicing excludes introns to generate mature mRNAs. CircRNAs can be divided into four subgroups according to different combinations of sequences and domains as follows: exonic circRNAs (ecircRNAs), which only contain exons; the circRNAs from introns, which only consist of introns (ciRNAs); and exon-intron circRNAs (eicircRNAs), which consist of both exons and introns (Zhang et al., 2013, 2014; Ashwal-Fluss et al., 2014; Li et al., 2015; Starke et al., 2015; Figure 1). Additionally, tRNA intronic circRNAs (tircRNAs) are generated during the pre-tRNA splicing process (Schmidt et al., 2019). EcircRNAs are the most common form of circRNAs and are derived from a unique back-splicing process that covalently attaches the 3′ splice acceptor to the downstream 5′ splice donor, forming a closed-loop structure (Figure 1).

Generally, three models have been proposed to illustrate the mechanisms of ecircRNA formation (Ashwal-Fluss et al., 2014; Zhang et al., 2014; Barrett et al., 2015; Ivanov et al., 2015). The connection of introns tends to form ciRNA after the generation of intronic lariat structures during internal reverse splicing (Zhang et al., 2013). Under some circumstances, if the intron sequences between the splice donor and the splice acceptor are retained in the canonical splicing process, the cyclizing transcripts form eicircRNAs (Li et al., 2015; Kristensen et al., 2019). The biogenesis of circRNAs is depicted in Figure 1.

Several characteristics of circRNAs have been identified with the deepening and widening relevant research. (1) Stability: In contrast to linear RNAs, circRNAs have single-stranded, covalently closed-ring structures without either 5′ caps or 3′ poly-A tails and, thus, are insusceptible to exonucleases. Therefore, most circRNAs exhibit a longer half-life than their linear counterparts. (2) Abundance: Generally, the expression of most circRNAs is lower than that of linear RNAs, but in different developmental phases, circRNAs are more abundant than the linear isoforms and can reach levels up to10-fold to 20-fold that of their linear isoforms (Xia et al., 2017). In humans, circRNAs are extensively expressed in the majority of tissues and are especially enriched in the neural system (Jeck et al., 2013; Rybak-Wolf et al., 2015). (3) Conservation: High-throughput sequencing and bioinformatic analyses have revealed that several circRNAs are highly conserved among species (Memczak et al., 2013; Rybak-Wolf et al., 2015), and only a small number of circRNAs are not evolutionarily conserved (AbouHaidar et al., 2014). Additionally, it has been found that 2,121 human circRNAs also map to the murine genome (Jeck et al., 2013). (4) Specificity: Although some circRNAs are conserved across species, the majority of circRNAs exhibit cell-specific, tissue-specific, and dynamic developmental-phase expression patterns (Salzman et al., 2013; Venø et al., 2015; You et al., 2015).

## Functions of Circular RNAs

### Circular RNAs Regulate Gene Splicing of Pre-RNA and Transcription

Circular RNAs have been demonstrated to regulate gene transcription *via* different mechanisms. EcircRNAs can regulate the linear isoform splicing of their parental genes by competing for splice sites. Ashwal-Fluss et al. (2014) revealed that circMbl originated from the second exon of the splicing factor *muscleblind* (*MBL*) and could compete with linear *MBL* mRNA for splicing. Interestingly, due to circMbl binding sites in the MBL protein, MBL could closely bind to circMbl and promote circMbl generation. Therefore, circMbl expression was positively upregulated by MBL expression and decreased the production of linear mRNA (Ashwal-Fluss et al., 2014).

Furthermore, circRNAs can bind to proteins to regulate the transcription of their locus genes. For instance, the two eicircRNAs circ-EIF3J and circ-PAIP2 could combine with the U1 small nuclear ribonucleoprotein (snRNP), forming EIciRNA–U1 complexes to further interact with polymerase II (Pol II) in the promoter regions of host genes and enhance the expression of their parental genes (Li et al., 2015). Additionally, ciRNAs such as circAankrd52 and ci-sirt7 can also regulate the transcription of their parental genes through *cis*-regulation of RNA Pol II (Zhang et al., 2013).

### Circular RNAs Function as MicroRNA Sponges

Accumulating evidence has proven that several circRNAs are rich in miRNA response elements (MREs) and act as miRNAs that can competitively bind other miRNAs and serve as intracellular competitive endogenous RNAs (ceRNAs), eliminating the suppressive effect of miRNAs on target genes (Hansen et al., 2013). miRNAs are another subtype of ncRNAs of approximately 22 nt length and play a pivotal role in posttranscriptional regulation by binding to specific sites in mRNA 3′-untranslated regions (Bartel, 2004; Trujillo et al., 2010). Recently, multiple lines of evidence have verified that some circRNAs are able to serve as miRNA sponges, including CDR1as or ciRS-7 (Memczak et al., 2013), circSRY (Hansen et al., 2013; You et al., 2015), circHIPK3 (Zheng et al., 2016), and many others.

### Circular RNAs Interact With RNA-Binding Proteins

Similar to their function as miRNA sponges, circRNAs can also interact with RNA-binding proteins (RBPs) and serve as protein baits or antagonists. For instance, circRNAs originating from *Foxo3* (circFoxo3) interact with cyclin-dependent kinase 2 (CDK2) and cyclin-dependent kinase inhibitor 1 (p21), thereby playing an anti-oncogenic role and blocking the cell cycle (Du et al., 2016). Other examples, including circMbl [33], circDNMT1 (Du et al., 2018), circAmotl1 (Yang et al., 2017), and circPABPN1 (Abdelmohsen et al., 2017, 1), primarily interact with RBPs. RBPs also regulate the production of circRNAs. Genome-wide siRNA screening will identify several RBPs as effective regulators. However, the specific mechanism by which RBPs regulate circRNAs still needs to be investigated.

### Circular RNAs Are Translated Into Proteins

Circular RNAs were initially regarded as ncRNAs due to their lack of a 5′ cap, a 3′poly-A tail, and translation initiation structures (Wilusz, 2018). However, convincing evidence has proven that some endogenous circRNAs can be translated into proteins.

The first circRNA found to be translated into protein was the genome of the hepatitis D virus, which consists of a single-stranded circRNA that encodes a hepatitis D antigen (Kos et al., 1986). Moreover, Legnini et al. (2017) reported that circZNF609 could be translated into a protein and, thus, controlled myoblast proliferation in mice when driven by an internal ribosome entry site (IRES). Additionally, Pamudurti et al. (2017) revealed that circMbl could be translated into protein in a cap-independent manner in fly heads. Analogously, circSHPRH (Zhang et al., 2018) and circFBXW7 (Yang Y. et al., 2018) were found to encode proteins capable of repressing human glioma tumorigenesis.

## Circular RNAs and Bladder Cancer

Accumulating evidence has proven that circRNAs are aberrantly expressed in many malignancies, including BCa. Advances in the elucidation of the pathogenesis of BCa have identified an abundance of dysregulated circRNAs in BCa cells and tissues. These aberrantly expressed circRNAs eventually lead to abnormalities in biological, genetic, and epigenetic information. Moreover, an increasing number of studies have shown that circRNAs play a pivotal role in the tumorigenesis of BCa, indicating that circRNAs could be targeted for diagnostic and therapeutic purposes. Here, we have summarized the circRNAs that are dysregulated in BCa (Table 1).

**TABLE 1:** Overview of dysregulated circRNAs in BCa.

CircRNAs	Dysregulation	miRNA	Implicated molecules and pathways	Functions	Validated samples	Clinicopathological relation	PMID
circZKSCAN1	Down	miR-1178-3p	p21	Suppresses proliferation, migration, invasion, and cell cycle, induces apoptosis	68 pairs and 137 BCa	T stage, grade, and lymphatic metastasis	31481066
circITCH	Down	miR-17/miR-224	p21/PTEN	Suppresses proliferation, migration, invasion, and cell cycle, induces apoptosis	72 pairs and 72 BCa	Grade	29386015
circRGNEF	Up	miR-548	KIF2C	Promotes cell proliferation, migration, invasion, and cell cycle	90 pairs	T stage and lymphatic metastasis	32305958
circINTS4	Up	miR-146b	NF-κB/P38/MAPK	Promotes proliferation, invasion, migration, and cell cycle, suppresses apoptosis	40 pairs	/	30723269
circVANGL1	Up	miR-605-3p	VANGL1	Promotes proliferation, cell cycle, invasion, and migration	87 BCa and 37 NC	T stage and metastasis	30146736
circMTO1	Down	miR-221	E-cadherin/N-cadherin	Suppresses cell proliferation, migration, and invasion	117 pairs	Lymphatic metastasis	30551873
circRIP2	Down	miR-1305	TGFβ2/Smad3	Suppresses proliferation, invasion, and migration	45 pairs and 58 BCa	T stage, grade, and metastasis	32019579
circ5912	Down	/	MET/TGF-β2	Suppresses proliferation, migration, and invasion	45 pairs and 58 BCa	T stage and lymphatic metastasis	31808751
circRIMS1	Up	miR-433-3p	CCAR1/Wnt	Promotes cell proliferation, migration, and invasion	20 pairs	T stage and grade	33230478
circFUT8	Down	miR-570-3p	KLF10/Slug	Suppresses migration and invasion	145 BCa	Grade and lymphatic metastasis	32072011
circHIPK3	Down	miR-558	MMP-9/VEGF	Suppresses migration, invasion, and angiogenesis	44 pairs	Grade, invasion, and lymphatic metastasis	28794202
circACVR2A	Down	miR-626	EYA4	Suppresses proliferation, migration, and invasion	50 pairs and 140 BCa	T stage, grade, and lymphatic metastasis	31101108
circSLC8A1	Down	miR-130b/miR-494	PTEN	Suppresses proliferation, migration, and invasion	70 pairs	T stage and grade	31228937
circFOXO3	Down	MiR-9-5p	TGFBR2	Suppresses proliferation, migration, and invasion	49 pairs	T stage and lymphatic metastasis	32612392
circ0067934	Up	miR-1304	Myc	Promotes proliferation, migration, and invasion	54 BCa	T stage and lymphatic metastasis	32346439
circUBXN7	Down	miR-1247-3p	B4GALT3	Suppresses proliferation, migration, and invasion	30 pairs and 54 BCa	T stage and grade	30312173
circVANGL1	Up	miR-1184	IGFBP2	Promotes cell proliferation, migration, and invasion	60 pairs	/	31758655
circBC04820	Up	miR-1184	ITGA3	Promotes cell proliferation, migration, and invasion	30 pairs	T stage and grade	32789658
circFAM114A2	Down	miR-762	ΔNP63	Suppresses the migration, invasion, and proliferation	31 pairs	T stage and grade	31969560
hsa_circ_0017247	Up	/	Wnt/β-catenin	Promotes proliferation, invasion, migration, and cell cycle, suppresses apoptosis	50 pairs	/	32096177
circ_100146	Up	miR-149-5p	RNF2	Promotes proliferation, invasion, and migration	68 pairs	T stage, lymphatic metastasis, grade, and tumor size	33149615
circZFR	Up	miR-377	ZEB2	Promotes proliferation, invasion, and migration	104 pairs	T stage, grade, and lymphatic metastasis	31746333
circ_0058063	Up	miR-486-3p	FOXP4	Promotes proliferation, invasion, and migration, suppresses apoptosis	94 pairs	T stage and metastasis	32181485
circFNDC3B	Down	miR-1178-3p	G3BP2/SRC/FAK	Suppresses cell proliferation, migration, and invasion	56 pairs	Grade, T stage, and lymphatic metastasis	30458784
hsa_circ_0091017	Down	miR-589-5p	/	Suppresses proliferation, migration, and invasiveness	40 pairs	/	31957821
circCEP128	Up	miR-145-5p	MYD88/MAPK	Promotes cell proliferation, invasion, and cell cycle, suppresses apoptosis	40 pairs	T stages, lymphatic metastasis, and differentiation	30939216
hsa_circ _0137439	Up	miR-142-5p	MTDH	Promotes cell proliferation, migration, and cell cycle	116 BCa, urine and 30 NC	T stage, grade, and lymphatic metastasis	31777254
circNR3C1	Down	miR-27a-3p	cyclin D1	Suppresses proliferation and cell cycle	44 pairs	/	31255724
circNR3C1	Down	/	BRD4/C-myc/EZH2	Suppresses proliferation, invasion, and cell cycle	54 BCa	/	33230453
circSEMA5A	Up	miR-330-5p	ENO1/SEMA5A	Promotes proliferation, invasion, and angiogenesis, suppresses apoptosis	50 pairs	T stage and tumor size	33176280
hsa_circ_0000144	Up	miR-217	RUNX2	Promotes cell proliferation, invasion, and cell cycle	21 pairs	/	30098434
circCDYL	Down	/	C-MYC	Suppresses cell proliferation, migration, and cell cycle	30 pairs	T stage	30968727
ciRs-6	Down	miR-653	March1	Suppresses proliferation and cell cycle and induces apoptosis	45 pairs and 58 BCa	T stage and grade	31819015
hsa_circ_0001944	Up	miR-548	PROK2	Promotes proliferation, invasion, apoptosis, and cell cycle	90 pairs	T stage, grade, lymphatic metastasis, and tumor size	32928266
circCEP128	Up	miR-145-5p	SOX11	Promotes proliferation and cell cycle but suppresses cell apoptosis	10 pairs	T stage, lymphatic metastasis, and tumor size	30134837
circ_0058063	Up	miR-145-5p	CDK6	Promotes cell proliferation, migration, and cell cycle, suppresses apoptosis	25 pairs	/	30362519
circRNA-3	Down	miR-182-5p	p27	Suppresses proliferation and cell cycle	47 pairs	/	30285878
circPRMT5	Up	miR-30c	SNAIL1/E-cadherin	Promotes invasion and migration	119 pairs	T stage and lymphatic metastasis	30305293
circ_103809	Up	miR-516a-5p	FBXL18	Promotes cell proliferation, migration, and gemcitabine chemoresistance	55 pairs	/	32922071
circFNTA	Up	miR-370-3p	FNTA/KRAS	Promotes invasion and cisplatin chemoresistance	23 pairs	/	32052578
circPDSS1	Up	miR-16	/	Promotes proliferation, invasion, and migration	72 pairs,	T stage	31868205
circ_0061140	Up	miR-1236	/	Promotes cell proliferation and invasion	42 pairs	lymphatic metastasis	32495864
circ_40365	Up	/	LDHA	Promotes cell proliferation, migration, and aerobic glycolysis	123 pairs	T stage, lymphatic metastasis, and tumor size	31814891
circPICALM	Down	miR-1265	FAK	Inhibits invasion	40 pairs and 128 BCa	T stage, grade, and lymphatic metastasis	31648990
circ_0006332	Up	miR-143	MYBL2	Promotes cell proliferation and invasion	32 pairs	T stage, lymphatic metastasis, and muscular invasion	31756170
circTFRC	Up	miR-107	TGFβ	Promotes proliferation and invasion	57 pairs	T stage, grade, and lymphatic metastasis	30782157
hsa_circ_0068307	Up	miR-147	c-Myc	Promotes cell proliferation and migration	30 pairs	/	32398967
hsa_circ_0001361	Up	miR-491-5p	MMP9	Promotes invasion and metastasis	69 pairs	T stage, grade, vessel and muscle invasion	31705065
circMYLK	Up	miR-29a	VEGFA/VEGFR2	Promotes cell proliferation, angiogenesis, and metastasis	32 pairs	T stage and lymphatic metastasis	28687357
hsa_circ_0068871	Up	miR-181a-5p	STAT3	Promotes cell proliferation and migration but suppresses apoptosis	44 pairs	T stage and lymphatic metastasis	30999937
circBPTF	Up	miR-31-5p	RAB27A	Promotes cell proliferation and migration	72 pairs	Grade and recurrence	30103209
circ0001429	Up	miR-205-5p	vegfa	Promotes cell proliferation and migration but suppresses apoptosis	20 pairs	/	30909190
circPTPRA	Down	miR-636	KLF9	Suppresses cell proliferation	64 pairs and 104 BCa	T stage and size	31821171
circ_0071196	Up	miRNA-19b-3p	CIT	Promotes cell proliferation and migration	80 BCa and 30 NC	/	32705161
circKIF4A	Up	miR-375/1231	NOTCH2	Promotes cell proliferation, migration, and invasion	50 pairs	/	32457613
circDOCK1	Up	miR-132-3p	Sox5	Promotes proliferation and migration	23 BCa and 32 NC	/	30983072
circEHBP1	Up	miR-130a-3p	TGFβR1/VEGF-D	Promotes lymphangiogenesis and lymphatic metastasis	186 BCa	Lymphatic metastasis and grade	33545359
circRBPMS	Down	miR-330-3p	RAI2	Suppresses cell proliferation and migration, represses cell cycle	90 pairs	Tumor size, clinical stage, lymphatic metastasis, and muscle invasion	33614236

*BCa, bladder cancer; circRNA, circular RNA; NC, normal control; T, tumor.*

### Expression Profile of Circular RNAs in Bladder Cancer

BCa is the most frequent urologic malignancy with high morbidity and mortality worldwide. Patients with advanced BCa have a poor prognosis and limited benefit from combination chemotherapy. Underlying oncogenesis at the molecular level is critical for the development of novel effective therapies for BCa. In the post-genome-sequencing era, a growing body of evidence suggests that circRNAs have essential roles in the initiation, development, and metastasis of BCa.

The first report of aberrant circRNA expression profiles in BCa was published in 2016 (Huang et al., 2016; Zhong et al., 2016). Zhong et al. (2016) found that 469 circRNAs were aberrantly expressed in BCa by using a circRNA microarray. Among these circRNAs, the expression of 285 was significantly upregulated, and the expression of 184 was downregulated in BCa tumors compared with their expression in paired adjacent normal tissues (Zhong et al., 2016, 25). Using RNA-seq to profile three matched BCa samples, Li et al. (2017) detected a total of 16,353 circRNAs, of which the expression of 47 circRNAs was upregulated, and the expression of 524 was downregulated more than 2-fold. Subsequently, genome-wide circRNA expression signatures have been shown to precisely and rapidly identify dysregulated circRNAs in BCa. These high-throughput sequencing results strongly indicate an important involvement of circRNAs in the pathogenesis of BCa, although their detailed biological functions and underlying mechanisms remain to be elucidated.

### Circular RNAs as Diagnostic and Prognostic Markers in Bladder Cancer

Although most patients with BCa can be treated with a multimodal strategy involving surgery with intravesical or systemic therapy, many patients are diagnosed at an advanced stage of disease due to lack of specific early diagnostic biomarkers and the optimal window for surgical intervention is missed. Cystoscopy is the gold standard tool for the diagnosis of BCa, but this invasive examination is painful. Therefore, the identification of effective and reliable biomarkers for BCa is urgently needed. Advances in sequencing technologies and bioinformatics have identified disease-related circRNAs in human saliva, plasma, urine, and other body fluids (Jafari Ghods, 2018; Vea et al., 2018; Kölling et al., 2019). Notably, the expression of circRNAs significantly differs between tumor samples and adjacent normal tissue in a number of solid malignancies (Geng et al., 2018); thus, these specifically expressed circRNAs are potential markers for cancer diagnosis and prognosis.

CircRNA molecules have great potential as liquid biopsy biomarkers of BCa due to their stability, long half-life, tissue specificity, and abundance. Additionally, prognosis prediction plays an important role in risk stratification and personalized management to prolong the life span of patients with BCa. Moreover, increasing evidence suggests that specific circRNAs may serve as diagnostic and prognostic biomarkers of BCa. These circRNAs are listed in Table 2 (diagnostic biomarkers) and Table 3 (prognostic biomarkers).

**TABLE 2:** The potential circRNA as diagnostic biomarkers in bladder cancer.

CircRNA	Dysregulation	Samples	AUC	Sensitivity	Specificity	Clinicopathological	PMID
				(%)	(%)	association	
hsa_circ_0018069	Down	Tissue	0.709	97.6	46.3	T stage, grade, and muscular invasion depth	30984788
circASXL1	Up	Tissue	0.77	68.6	76.9	T stage, grade, and lymphatic metastasis	31966702
hsa_circ_0077837	Down	Tissue	0.775	–	–	Grade, invasion, and lymphatic metastasis	32250047
hsa_circ_0004826	Down	Tissue	0.79	–	–	Grade, invasion, and lymphatic metastasis	32250047
circZFR	Up	Tissue	0.8216	–	–	T stage, grade, and lymphatic metastasis	31746333
circ0006332	Up	Tissue	0.86	80.2	86	T stage, lymphatic metastasis, and muscular invasion	31756170
circPRMT5	Up	Tissue, serum, and urine	–	–	–	T stage, lymphatic metastasis	30305293
circCEP128	Up	Tissue and blood	–	–	–	T stages, lymphatic metastasis, differentiation	30134837
hsa_circ _0137439	Up	Urine	0.89	87.93	80.06	T stage, grade, and lymphatic metastasis	31777254

*AUC, area under the curve; circRNA, circular RNA.*

**TABLE 3:** The potential circRNAs as prognostic biomarkers in bladder cancer.

CircRNA	Dysregulation	Prognosis	Univariate Analysis			Multivariate Analysis			PMID
			HR	95% CI	p	HR	95% CI	p	
circLPAR1	Down	DSS	0.364	0.1970–00.673	0.001	0.364	0.1970–00.673	0.001	30867795
circASXL1	Up	OS	4.831	1.718–13.514	0.007	2.214	1.002–5.647	0.046	31966702
circRIP2	Down	OS	0.339	0.128–0.898	0.029	0.332	0.122–0.904	0.031	32019579
circPICALM	Down	OS	0.274	0.150–0.501	0	0.443	0.234–0.841	0.013	31648990
circ_403658	Up	OS	3.21	–	0.014	4.04	–	0.022	31814891
circHIPK3	Down	DFS	4.315	1.597–8.221	0.006	3.364	1.297–8.768	0.007	32194801
hsa_circ_0077837	Down	OS	0.13	0.03–0.50	0.003	0.352	0.15–0.82	0.016	32250047
		RFS	0.192	0.06–0.63	0.006	0.232	0.06–1.07	0.045	32250047
circEHBP1	Up	OS	2.052	1.337–3.152	0.001	1.808	1.151–2.841	0.01	33545359
		DFS	1.632	1.114–2.391	0.001	1.555	1.042–2.320	0.031	33545359

*CI, confidential interval; circRNA, circular RNA; DFS, disease-free survival; DSS, disease-specific survival; HR, hazard ratio; OS, overall survival; RFS, recurrence-free survival.*

Li et al. (2019a) recently showed that circ0006332 expression was significantly upregulated in BCa tissue compared with adjacent non-tumorous tissue. Elevated circ0006332 was positively correlated with tumor stage, lymphatic metastasis, and muscular invasion. Importantly, circ0006332 showed a high area under the curve (AUC) of 0.86, a sensitivity of 80.2%, and a specificity of 86.0%. This study indicated that circ0006332 might be a reliable diagnostic biomarker for BCa. Additionally, Tang et al. (2017) analyzed the expression level of circASXL1 in cancer and adjacent para-cancerous tissue. CircASXL1 expression was prominently upregulated in BCa tissues. When distinguishing BCa tissues from normal tissues, the AUC of circASXL1 was 0.77, showing a sensitivity of 68.6% and a specificity of 76.9%. In addition, Kaplan–Meier survival analysis suggested that patients with BCa with upregulated circASXL1 expression had significantly reduced overall survival (OS). Further univariate and multivariate Cox regression also indicated that the circASXL1 expression level was an independent risk factor for OS after radical cystectomy.

In addition, Shen et al. (2020) analyzed the expression of hsa_circ_0077837 and hsa_circ_0004826 in cancer and adjacent para-tumorous tissues. The results showed that both the hsa_circ_0077837 and hsa_circ_0004826 expression levels were significantly downregulated in BCa tissues (Shen et al., 2020, 0004826). Moreover, the reduced expression of hsa_circ_0077837 and hsa_circ_0004826 was significantly associated with worse clinicopathological features. The AUC of hsa_circ_0077837 and hsa_circ_0004826 was 0.775 and 0.79, respectively. Consistently, they also demonstrated that patients with BCa positive for hsa_circ_0077837 and hsa_circ_0004826 had more prolonged relapse-free survival (RFS) and OS. The Cox multivariate analysis indicated that the expression levels of these two circRNAs might serve as independent prognostic predictors of RFS and OS.

Furthermore, the expression of hsa_circ_0018069 was found to be significantly downregulated in BCa tissue and cell lines (Li et al., 2019b, 0018069). The decreased expression of hsa_circ_0018069 was associated with some of clinicopathological features of BCa. The AUC was 0.709, and the sensitivity and specificity were 97.6% and 46.3%, respectively. Additionally, Zhang et al. (2019) verified the expression of circZFR in a cohort of 104 patients with BCa. Its expression was significantly increased in tumor tissues compared with paired adjacent non-tumor samples and had good clinical diagnostic value based on the receiver operating characteristic (ROC) curve with an AUC value of 0.8216. These studies showed the diagnostic potential of circRNAs. CircRNA signature expression analysis may have better performance and increased potential for BCa diagnostics than analysis of a single circRNA candidate; however, this avenue has not yet been explored for BCa. The limitation of using tissue for diagnosis is the invasive nature of sample collection, and it is typically collected at the time of surgery when the diagnosis is already confirmed.

Chen et al. (2018) reported that circPRMT5 was significantly overexpressed in BCa tissues, serum, and urine compared to normal controls. Additionally, clinical analysis revealed that the high expression of circPRMT5 was positively associated with lymphatic metastasis and tumor progression in patients with BCa. CircPRMT5 could be used as a novel biomarker for the diagnosis and prognosis of BCa. Sun et al. (2019b) assessed the expression of circCEP128 in a cohort of 40 patients with BCa in both tissues and blood samples, and its expression was significantly increased in tumor samples compared with matched adjacent non-tumor samples. Significantly, circCEP128 levels were associated with various clinical characteristics, including tumor stage, lymphatic metastasis, and differentiation. Taken together, these data prove that circCEP128 is a promising biomarker for BCa that can be measured non-invasively. Clinically, the diagnosis of BCa mainly is based on invasive cystoscopy, which is painful and sometimes associated with adverse effects, such as urinary tract infection, frequent urination, and visible hematuria. Thus, detection of alterations in the levels of circRNAs in the serum and urine could be utilized as a diagnostic tool for BCa instead of the use of tissue samples. These new biomarkers may receive enormous attention and interest due to non-invasiveness and inexpensiveness.

In addition, hsa_circ_0137439 expression was found to be remarkably increased in BCa tissue and cell-free urine (Song et al., 2020). Intriguingly, urinary cell-free hsa_circ_0137439 expression could serve as a diagnostic biomarker for BCa with an AUC value of 0.890, sensitivity of 87.93%, and specificity of 80.06% at the cutoff value of 1.360. Moreover, urinary hsa_circ_0137439 expression could also distinguish NMIBC from MIBC with an AUC value of 0.798, a sensitivity of 88.56%, and a specificity of 73.45% at a cutoff value of 0.628. Additionally, BCa patients with elevated levels of hsa_circ_0137439 in urine had significantly worse OS and RFS, indicating that this circRNA is a potential diagnostic and prognostic biomarker of BCa that can be measured non-invasively. These studies highlight the potential of urine or serum assessment of circRNAs; however, crucial methodological information was not reported, and none of these investigations mentioned the quality or quantity of total RNA collected from urine or blood samples for analysis or other validation techniques. In addition, these circRNAs are differentially expressed in BCa and normal bladder tissues, but most of them are undetectable in urine or serum.

The prognostic potential of circRNAs is of great interest, as circRNAs play an important role in tumorigenesis of BCa. A study of 168 patients with BCa showed that circPICALM expression was negatively associated with various clinical characteristics (Yan et al., 2019). Kaplan–Meier survival analysis showed that patients with a lower expression of circPICALM had poorer outcomes. Further univariate and multivariate Cox regression indicated that circPICALM expression was an independent prognostic factor for OS for patients with BCa. Moreover, circHIPK3 expression was prominently downregulated in BCa tissues and was negatively correlated with several clinical characteristics, and patients with low-level expression of circHIPK3 had worse DFS rates (Xie et al., 2020b). In addition, Su et al. (2020) demonstrated that circRIP2 could accelerate BCa progression, and upregulated circRIP2 expression was significantly correlated with aggressive clinicopathological characteristics. The survival analysis suggested that patients with BCa with high circRIP2 levels had significantly reduced OS (Su et al., 2020). Additionally, Zhu et al. (2021) reported that circEHBP1 expression was significantly downregulated in BCa tissues. They also demonstrated that patients with low levels of circEHBP1 had worse DFS and OS. Wei et al. (2019) demonstrated that circ_403658 expression was upregulated in BCa under hypoxia. Moreover, clinical study showed that higher circ_403658 levels were associated with poorer survival outcomes.

Although tissue biopsy is essential for the diagnosis of BCa, it is an invasive and painful procedure for patients. Therefore, it is difficult to perform biopsy frequently. CircRNAs have been reported to be found in most types of body fluids and easy to collect. Urinary circRNAs reflect the current state of BCa in a real-time manner, as they are secreted by their parental cancer cells. For these reasons, urinary circRNAs allow to monitor the BCa with repeatedly and readily non-invasive evaluation, especially for routine follow-up visits. In addition, the combination of circRNAs would be valuable for establishing a useful panel for BCa management. Transurethral resection of bladder tumor (TURBT) is usually performed to diagnose MIBC in clinical practice. However, there is no biomarker for predicting MIBC before TURBT. In MIBC patients, the tumor cells may easily invade into blood and lymphatic vessels. Thus, the circRNAs related to BCa released into the circulatory system will also be increased. Therefore, the quantity of circRNAs from blood samples or ratio of urinary to blood samples would be a potential biomarker for predicting the invasive subtype of BCa. Based on the recent genome characterization of BCa, it is a heterogeneous disease and can be grouped into several molecular subtypes (Knowles and Hurst, 2015). Although, to date, there is no study to report the relationship between the characteristics of circRNAs and the molecular subtypes. The contents of urinary circRNAs can be influenced by parental BCa cells. Thus, they can also be potential candidates for predicting the molecular subtypes of BCa.

Basic research indicates that circRNAs have tremendous potential for diagnosing and predicting the prognosis of BCa. There are still many obstacles hindering the clinical use of circRNAs as biomarkers for BCa. Notably, more bench and clinical work should be carried out to screen out more reliable biomarkers for clinical application. Therefore, more studies of large cohorts are required to validate the actual diagnostic and prognostic effectiveness of circRNAs.

### Circular RNA Dysregulation in Bladder Cancer

**FIGURE 2:**
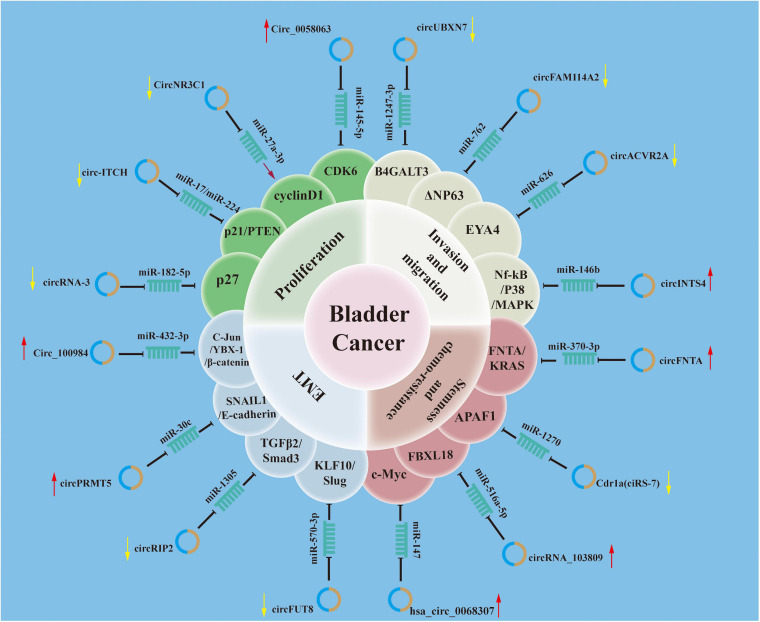
Roles of circular RNAs (circRNAs) in mechanisms mediating tumorigenesis and progression of bladder cancer. The schematic diagram summarizes the roles of circRNAs in the progression and involvement of circRNAs–microRNA (miRNA)–targets axis of bladder cancer.

Several pathways and proteins associated with the malignant features of BCa have been demonstrated to be dysregulated circRNAs. These circRNAs serve as oncogenes or tumor suppressors and affect cancer phenotype in various ways. Diverse mechanisms have been proposed for their functions in driving carcinogenesis, including increasing cell proliferation, promoting invasive and migratory capacity, enhancing endothelial–mesenchymal transition (EMT), sustaining stemness, and enabling chemotherapy resistance (Figure 2).

#### Increasing Proliferation

Near-limitless proliferation capacity is one of the most critical characteristic changes in tumor cells (Hanahan and Weinberg, 2011). The regulation of BCa cell proliferation is a complex and precise process involving various signals that drive cells to divide and grow by regulating the cell cycle. Consequently, dysregulation of the cell cycle plays a pivotal role in the excessive replication of tumor cells. Recently, a growing body of evidence has suggested that circRNAs can control tumor cell proliferation.

A variety of signaling pathways contribute to cell cycle regulation, including phosphoinositide 3-kinase (PI3K)/AKT and mitogen-activated protein kinase (MAPK), and there is evidence that these signaling pathways can be altered by circRNAs. Yao et al. (2020, 139) revealed that the expression of circZNF139, a circRNA derived from *ZNF139*, was significantly upregulated in BCa. Mechanistic studies revealed that circZNF139 activates the PI3K/AKT pathway and stimulates the proliferative potential of BCa cells. The researchers did not include xenograft models or patient tissue to verify these findings. The detailed mechanism of how circZNF139 regulates PI3K/AKT pathway was not explored. In addition, Sun et al. (2019b) reported that circCEP128 was overexpressed in BCa tissues and cells; knockdown of circCEP128 caused suppression of cell growth and mobility as well as cell cycle arrest. Furthermore, they reported that circCEP128 could act as a sponge of miR-145-5p; knockdown of circCEP128 expression decreased the proliferative effect of miR-145-5p in MAPK-dependent signaling pathway. Similarly, circINTS4 plays a similar role in BCa by competitively binding to miR-146b, thus regulating the downstream P38/MAPK pathway (Zhang et al., 2020, 4). Knocking down circINTS4 inhibited proliferation, invasion, and migration; induced G_1_/S cell cycle arrest; and dramatically promoted apoptosis. These results are interesting; most research has focused on overexpression, and it is difficult to explain how highly expressed circRNAs could be maintained in BCa cells that divide so frequently. Therefore, the concrete mechanisms of these circRNAs should be characterized in more detail.

Moreover, several circRNAs can regulate cell proliferation by targeting the pivotal molecules involved in cell cycle regulation. p21, also known as cyclin-dependent kinase inhibitor 1, is a negative modulator of the cell cycle that induces cell cycle arrest and blocks cell proliferation (Liu and Lozano, 2005). CircITCH can bind miR-17/miR-224, which results in increased levels of P21, thus suppressing BCa tumor cell proliferation (Yang C. et al., 2018). Bi et al. (2019), (1) studied the interaction and function of circZKSCAN1 and its downstream target miRNAs. They found that circZKSCAN1 downregulated p21 expression by sponging miR-1178-3p and suppressing proliferation. Moreover, P27, encoded by *CDKN1B (cyclin-dependent kinase inhibitor 1B)*, is often referred to as a negative regulator that arrests cell cycle progression at the G_1_/S phase (Coqueret, 2003). Additionally, Xie et al. (2018) revealed that circRNA-3 expression was significantly downregulated in BCa tissues. CircRNA-3, which can directly bind with miR-182-5p and inhibit its function, can upregulate P27 expression and induce cell cycle arrest at the G_0_/G_1_ phase in cancer cells. Similarly, Zheng et al. (2019) discovered that the expression of circNR3C1, a circRNA originating from *NR3C1*, was downregulated in BCa. Functionally, overexpression of circNR3C1 significantly halted cell cycle progression and inhibited the proliferation of BCa cells *in vitro* and *in vivo*. Mechanistically, circNR3C1 could effectively sponge miR-27a-3p to suppress the expression of cyclin D1 and induce cell cycle arrest at G_0_/G_1_ phase. Conversely, Sun et al. (2019a) demonstrated that circ0058063 was significantly overexpressed in BCa tissues. The knockdown of circ0058063 could impair tumor cell proliferation and migration and induce cell apoptosis by sponging miR-145-5p to upregulate CDK6 expression. Above all, these studies demonstrated that ectopic overexpression or silencing of these circRNAs could obviously inhibit cell proliferation and tumor growth.

An increasing number of studies have identified that more circRNAs could specifically sequester target miRNAs, thereby regulating downstream signaling pathways and genes involved in the proliferation of BCa cells (Huang et al., 2018; Su et al., 2019b; Sun J. et al., 2019; Zeng et al., 2019; Jin et al., 2020; Song et al., 2020, 0137439; Wang L. et al., 2020; Xie et al., 2020a; Yang C. et al., 2020). The studies described above showed that an increase in proliferation induced by circRNAs favored cancer cells. It might be essential to determine whether diluted circRNA level due to cell division in BCa cells may result in different proliferation rates.

#### Promoting Invasion and Metastasis

Metastasis is a multistep process involving invasion and migration, which are indispensable features of cancer. Both interruption of the epithelial barrier and disruption of the extracellular matrix permit cellular dissemination, synonymous with the characteristics of invasion and migration in malignancies (Hanahan and Weinberg, 2011).

The majority of circRNAs promote invasion and metastasis *via* circRNA–miRNA–mRNA interaction networks. Mounting evidence revealed that circRNAs can also play an oncogenic role in promoting invasion and metastasis of BCa cells. For instance, circ100984 was found to be highly expressed in BCa (Tong et al., 2020). Silencing circ100984 repressed BCa cell viability, invasion, and migration both *in vitro* and *in vivo*. Mechanistically, circ100984 acted as a ceRNA for miR-432-3p, which subsequently regulated the c-Jun/YBX-1/β-catenin loop. Similarly, Yang C. et al. (2020) reported that circRGNEF expression was upregulated significantly in BCa. A high level of circRGNEF was associated with aggressive phenotypes of BCa. Further investigation unveiled that silencing circRGNEF suppressed BCa cell invasion and metastasis by targeting the miR-548/KIF2C axis *in vitro* and *in vivo*.

A recent study reported that a novel circRNA (circ_0058063) increased forkhead box P4 (FOXP4) expression by inhibiting miR-486-3p in BCa cells (Liang et al., 2020). Silencing circRNA0058063 remarkably reduced BCa cell migration and invasion. These results indicated the existence of a novel circ_0058063/miR-486-3p/FOXP4 regulatory axis in BCa tumorigenesis and metastasis. Additionally, Jiang et al. (2020, 048201) identified that circBC048201 expression was abnormally upregulated in BCa tissues and cells. Mechanistic investigation revealed that interference with circBC048201 repressed BCa migration and invasion *via* the miR-1184/ITGA axis. Moreover, other circRNAs have been shown to play oncogenic roles in BCa to promote invasion and metastasis (Zeng et al., 2019; Zhang et al., 2019, 2020, 3; Wang F. et al., 2020; Wang H. et al., 2020, 2; Yang D. et al., 2020; Yao et al., 2020, 139).

Conversely, a number of circRNAs have been identified as tumor suppressors in BCa. For instance, reduced circFNDC3B expression in BCa was associated with aggressive clinical characteristics (Liu et al., 2018a). Functionally, overexpression of circFNDC3B significantly suppressed tumor invasion and metastasis. Mechanistically, circFNDC3B served as a miR-1178-3p sponge to suppress G3BP2, subsequently inhibiting the downstream steroid receptor coactivator (SRC)/focal adhesion kinase (FAK) signaling pathway. Similar to circFNDC3B, circUBXN7 expression was significantly downregulated in BCa tumors and cell lines (Liu et al., 2018b, 3). Overexpression of circUBXN7 significantly inhibited BCa cell migration and invasion by sponging miR-1247-3p, resulting in increased beta-1,4-galactosyltransferase 3 (B4GALT3) expression. Additionally, Liu et al. (2020) found that circFAM114A2 expression was dramatically downregulated in both BCa tissue and cell lines. Furthermore, they reported that circFAM114A2 increased the expression of △NP63 by sponging miR-762 to suppress the migration and invasion of BCa *in vitro*. Other regulatory cascades comprising circRNA, miRNA, and mRNA that are involved in regulating invasion and metastasis in BCa have been reported, including circHIPK3/miR-558/MMP-9/VEGF (Li et al., 2017), circRIP2/miR-1305/TGFβ2 (Su et al., 2020), circ-ITCH/miR-17/miR-224/p21/PTEN (Yang C. et al., 2018), circMTO1/miR-221/E-cadherin/N-cadherin (Li Y. et al., 2019), and circFUT8/miR-570-3p/KLF10/Slug (He Q. et al., 2020). Together, these findings indicate that circRNAs play important roles in invasion and metastasis in BCa. Therefore, it can be speculated that manipulation of these circRNAs associated with the EMT could suppress BCa progression and could be a potential therapeutic strategy.

#### Epithelial to Mesenchymal Transition

Epithelial-to-mesenchymal transition is a vital cell matrix-associated event contributing to carcinogenesis and metastasis. EMT is characterized by a loss of cell-to-cell adhesion and cell polarity, resulting in the development of motility, migratory, and invasive cellular characteristics. Several signaling pathways and protein biomarkers have been identified to be involved in this process (Lamouille et al., 2014). Here, we have summarized the roles of various circRNAs that have been reported to regulate EMT in BCa.

Chen et al. (2018) revealed that circPRMT5 expression was upregulated in BCa tissues compared with normal controls. They also found that knockdown of circPRMT5 increased the expression of epithelial marker E-cadherin but decreased the expression of mesenchymal markers vimentin and N-cadherin, indicating that circPRMT5 promotes EMT in BCa (Chen et al., 2018). A recent study reported that the expression of a novel circRNA, circPICALM, was downregulated in BCa tissues (Yan et al., 2019). Moreover, circPICALM acted as a sponge for miR-1265 and suppressed EMT *via* the miR-1265/STEAP4/FAK axis.

Additionally, Su et al. found that circTFRC expression was upregulated and contributed to aggressive BCa phenotypes and poor survival (Su H. et al., 2019). Mechanistically, they demonstrated that circTFRC acted as a ceRNA for miR-107. Functionally, EJ or T24 cells overexpressing circTFRC displayed EMT morphological changes. Meanwhile, knocking down circTFRC significantly increased E-cadherin expression. These data suggested that circTFRC could play an important role in driving EMT in BCa.

Moreover, Su et al. (2020) revealed that the expression of another novel circRNA, circRIP2, was significantly downregulated in BCa tissues and cell lines and was negatively associated with tumor stage, grade, and metastasis. Furthermore, they found that circRIP2 sponged miR-1305 to stimulate EMT *via* the transforming growth factor (TGF)β2/smad3 pathway. CircRIMS1 has been reported to be an oncogenic circRNA in BCa (Wang F. et al., 2020). miR-497-5p could be specifically and directly regulated by circRIMS1. Mechanistically, inhibition of circRIMS1 enhanced E-cadherin expression but suppressed the expression of N-cadherin and vimentin *via* the circRIMS1/miR-433-3p/CCAR1 regulatory axis. Collectively, upregulation of circRIMS1 expression could facilitate EMT in BCa. *In vitro* studies supported a relationship between dysregulated circRNAs and EMT in BCa, including circ0006332, circMTO1, circ5912, circFUT8, circ100984, and circRBPMS, along with upregulation of mesenchymal marker expression, including N-cadherin and vimentin (Li et al., 2019a; Li Y. et al., 2019; Su et al., 2019a; He Q. et al., 2020; Tong et al., 2020; Yang et al., 2021). These findings demonstrate that circRNAs may be therapeutic targets, and the development of tools such as CircInteractome (Dudekula et al., 2016) has improved the potential to develop siRNAs that are able to selectively inhibit circRNAs of interest. Our understanding of the molecular mechanisms of circRNA in BCa EMT is still very superficial. CircRNAs undoubtedly play an important role in EMT, and further research is urgently needed.

#### Stemness and Chemotherapy Resistance

Cancer stem cells are a subgroup of cancer cells that have tumor-initiating and self-renewal capabilities and are thought to be responsible for metastasis and treatment failure (Dalerba and Clarke, 2007). Growing evidence has revealed that circRNAs might be involved in the stemness of cancer cells.

Gu et al. (2018) identified that circGprc5a expression was upregulated in BCa. Moreover, elevated circGprc5a expression was related to clinical severity and progression. Mechanistically, circGprc5a had peptide-coding potential and function *via* the circGprc5a–peptide–Gprc5a axis in stemness. Hsa_circ_0068307 was shown to be overexpressed in human BCa cell lines (Chen Q. et al., 2020). Hsa_circ_0068307 silencing suppressed cell migration and proliferation in T24 and UMUC3 cells. Hsa_circ_0068307 was demonstrated to bind to miR-147 and functioned as a ceRNA to upregulate miR-147-targeted *c-myc.* Thus, hsa_circ_0068307 was hypothesized to support stemness properties *via* the miR-147/C-MYC axis. In these studies, circRNAs were revealed to promote self-renewal, which is a typical property of stemness. These circRNAs might be novel biomarkers of stemness in BCa. In addition, with appropriate techniques for silencing or overexpressing circRNAs, manipulating circRNA stemness *in vivo* may influence the response to chemotherapeutics.

Although chemotherapy improves survival time in BCa, its application is limited as cancer cells acquire resistance to agents used. Combinations of chemotherapeutic drugs offer limited benefits (Vasan et al., 2019). Recently, accumulating evidence has proven that several circRNAs play important roles in the acquired resistance of BCa to chemotherapy, including circELP3, circFNTA, circHIPK3, Cdr1as (ciRS-7), and circ_103809 (Su et al., 2019c; Yuan et al., 2019; Chen J. et al., 2020; Huang et al., 2020; Xie et al., 2020b).

CircHIPK3 expression was significantly downregulated in BCa tissues and negatively correlated with several clinical characteristics (Xie et al., 2020b). CircHIPK3 was expressed at low levels in gemcitabine-resistant cell lines, and overexpression of circHIPK3 decreased IC50 of gemcitabine and promoted cytotoxicity. In addition, Chen J. et al. (2020) identified a circRNA, circFNTA, regulated by the androgen receptor (AR), that increased the aggressive BCa phenotype and promoted cisplatin chemoresistance. Mechanistically, the AR suppressed the RNA-editing gene *ADAR2* by binding to the promoter region to increase circFNTA levels, which then sponged miR-370-3p to upregulate the expression of its host gene *FNTA*. This AR-mediated ADAR*2*/circFNTA/miR-370-3p/FNTA axis subsequently activated KRAS signaling to dysregulate cancer invasion and promote chemoresistance to cisplatin. Moreover, a study from Su et al. (2019c) revealed that knockdown of circELP3 with siRNA significantly decreased the sphere-forming capability of BCa cells under hypoxic conditions. Silencing circELP3 decreased the expression of stem cell markers Oct4 and Sox2, indicating that hypoxia-elevated circELP3 expression may facilitate cisplatin sensitivity by targeting cancer stem-like cells.

Cells overexpressing Cdr1as were thought to demonstrate increased cisplatin sensitivity as a result of regulation of the miR1270/APAF1 axis (Yuan et al., 2019). Conversely, circRNA_103809 silencing was associated with decreased gemcitabine resistance in EJ and T24 cells through targeting miR-516a-5p/FBXL18 signaling (Huang et al., 2020, 18). Additionally, circRNAs may act as biomarkers to predict patients’ response to chemotherapy, and these studies also raised the possibility that circRNAs could be therapeutic targets. In the above studies, silencing of upregulated circRNAs was able to reverse resistance to chemotherapeutic agents, and overexpression of downregulated circRNAs was able to promote chemotoxicity in resistant BCa cell lines. However, these findings were not validated across all the BCa cell lines investigated.

Overall, these studies illustrate that circRNAs have various significant regulatory functions in BCa tumorigenesis and progression.

## Future Perspectives and Therapeutic Potential of Circular RNAs in Bladder Cancer

Circular RNAs are being increasingly identified with the rapid advances in next-generation sequencing and bioinformatics. Although some circRNAs have diagnostic and prognostic potential, most circRNAs cannot be used for clinical applications because of their low sensitivity or specificity. More importantly, researchers have also considered utilizing the differentially expressed circRNAs in clinically useful samples other than BCa tissues, for example, blood and urine. CircRNAs in the blood and urine may offer a simple and non-invasive approach to diagnose BCa, facilitating clinical management, such as serum circCEP128 and urine circPRMT5 and hsa_circ _0137439 (Chen et al., 2018; Sun et al., 2019b; Song et al., 2020).

Systemic cisplatin-based cytotoxic chemotherapy has dramatically improved the outcome of patients with BCa. However, acquired resistance to chemotherapy is an intractable issue in BCa treatment. Recently, several circRNAs dysregulated in BCa were associated with enhanced chemotherapy resistance. One inspiring application is targeting circRNAs as an accurate and effective method for promoting chemotherapy sensitivity. As described above, circELP3 is upregulated in BCa. This upregulation leads to BCa progression and cisplatin resistance. Precise circELP3-specific RNA drugs may have potential applications in treating cisplatin resistance in BCa (Su et al., 2019c, 3). Depletion of circFNTA decreases the expression of its host gene FNTA, suppressing BCa cell invasion and increasing cisplatin sensitivity *in vivo* (Chen J. et al., 2020). Additionally, circRNA_103809 was highly expressed in BCa associated with a poor progression in BCa patients. Circ_103809 can promote cell proliferation and migration by sponging miR-516a-5p and promoting FBXL18 expression. Silencing of circ_103809 increased the sensitivity of BCa cells to gemcitabine treatment (Huang et al., 2020, 103809). On the other hand, owing to their stability and multiple binding sites for miRNAs, tumor suppressor circRNAs are potential therapeutic sponge vectors to sensitize chemotherapy. For example, circHIPK3 alleviates gemcitabine resistance in BCa (Xie et al., 2020b, 3). CiRS-7 might sponge miR-1270 to increase cisplatin sensitivity of BCa cells (Yuan et al., 2019). The sponging feature of tumor suppressor circRNAs might be adopted to design specifically targeting corresponding oncogenic miRNAs, subsequently playing a therapeutic role. A main challenge to impede any of the abovementioned strategies will be precise transport into the cancer cells to avoid off-target effects. Although studies on chemoresistance in BCa remain nascent, comprehensive elucidation of circRNA mechanisms will advance our efforts to address the problem.

Accumulating evidence indicates that circRNAs play a pivotal role in the development of BCa, such as cell proliferation, invasion, migration, and metastasis, offering possibilities for circRNAs as potential therapeutic targets. Suppressing circRNA expression to regulate the corresponding miRNA expression and subsequently regulate downstream mRNAs may be a promising therapeutic strategy for BCa. Several technologies have been developed to target circRNAs for therapeutic purposes. CircRNAs could be typically knocked down by RNA interference using short hairpin RNA (shRNA) or short interfering RNA (siRNA). To knock down oncogenic circRNA without affecting the corresponding parental linear mRNA, the unique back-spliced junction (BSJ) site is specifically targeted. However, there are some challenges with RNA interfering, including immunogenicity, low *in vivo* delivery efficiency, quick degradation, and off-target effects (Pai et al., 2006). Furthermore, the clustered regularly interspaced short palindromic repeats-caspase 9 (CRISPR-Cas9) system, a revolutionary genome-editing method, has also shed light on circRNA-based therapy. However off-target effect is the main bottleneck of CRISPR-Cas9, which could result in undesired genetic alteration. More importantly, CRISPR/Cas13-mediated circRNA could knock down circRNAs specifically and efficiently. This system could be used as an effective tool to directly target oncogenic circRNAs in a specific and robust manner, and they provide a solid basis for therapeutic potential (Koch, 2021).

Conversely, restoring circRNA expression *via* engineered circRNAs that target dysregulated miRNAs may suppress aggressive phenotypes and progression (Rossbach, 2019). Therefore, artificially synthesized tumor suppressor circRNAs are inspiring therapy targeting oncogenic miRNA. These synthesized molecules seem to be the ideal inhibitors for oncogenic miRNA, as they can be designed to have multiple binding sites for the unique miRNA. Recently, pioneer studies have shown that artificial circRNAs can be stably expressed and function efficiently as miRNA sponges in digestive tract cancers (Liu X. et al., 2018; Wang et al., 2019). A similar strategy may also be used for BCa treatment. Besides, as circRNAs have a longer half-life, they can be used at a lower dose and frequency.

Importantly, more studies are needed to figure out how to efficiently deliver circRNAs to cells to regulate cancer progression without immunogenicity and with sustained long-term effect. As nanoparticle delivery has the abilities of resisting degradation, facilitating cell engulfment, and avoiding immune activation, they could be used to deliver circRNA expression vectors. Recently, several studies reported that nanoparticle delivery of circRNA plasmids can suppress the phenotypes of tumor *in vivo* (Du et al., 2017a; Lu et al., 2020). In addition, exosomal circRNAs modulated the progression of cancer, remodeling of the tumor microenvironment, immune response, recurrence, and metastasis. Thus, exosome delivery of engineered circRNAs that produce specific therapeutic proteins into cancer cells is another inspiring research field (Bei et al., 2018).

Besides, there are several gaps of circRNAs between the benchwork and clinical application, which need to be fulfilled in the future. The mechanisms involved in circularization and degradation remain elusive. Precise degradation of overexpressed circRNAs is an ideal approach to treat cancer. Additionally, there are multiple dysregulated circRNAs in the same cancer tissue, and circRNA-mediated molecular signaling pathways have crosstalks between each other. It is not clear whether they affect each other.

Overall, current mechanistic and clinical insights highlight the pivotal roles of circRNAs in BCa, but we believe that these works are still at the nascent stage. The deeper understanding of circRNA modulating mechanisms, downstream regulatory networks, and clinical relevance will increase our understanding of the roles of circRNAs and development of circRNA-based diagnosis, prognosis, and armamentarium for BCa.

## Conclusion

Over the past decades, circRNAs have been identified as “by-products” or “dark matters.” Recently, the mystery of circRNAs has gradually been uncovered owing to advances in sequencing technologies and bioinformatics. As a type of illness with high morbidity and mortality, BCa is a serious threat to human health. Recently, circRNAs have been demonstrated to be involved in the tumorigenesis of BCa.

As mentioned above, a large amount of research has shown that circRNAs are differentially expressed between BCa tissues and adjacent normal tissues. Notably, circRNAs have been discovered to be significantly associated with clinicopathologic features of BCa. These findings represent a plethora of new opportunities to explore novel diagnostic and prognostic biomarkers for BCa. Intriguingly, circRNAs in body fluids may function as non-invasive biomarkers for BCa, such as circCEP128, circPRMT5, and hsa_circ _0137439. As summarized in this review, circRNAs play pro-cancer and anti-cancer or chemotherapy sensitivity-regulating roles in the carcinogenesis, progression, and drug resistance of BCa. Characterization of the mechanisms by which these dysregulated circRNAs that process them contribute to BCa offers opportunities for therapeutic intervention. For example, targeting circRNAs that are involved in chemotherapy resistance may promote sensitivity to cisplatin or gemcitabine treatment, such as circELP3, circFNTA, and circ103809. Additionally, the major mechanism of circRNAs exerting functions in BCa is serving as miRNA sponges to target downstream genes, while other potential molecular mechanisms are limited and warrant further investigation.

Ultimately, our growing knowledge on circRNA transportation, localization, degradation, and interactome will shed light on potential therapeutic targets and tremendously benefit BCa patients in the near future.

## Author Contributions

FC, ZN, and WH did the conceptualization. FC, BZ, ZN, and WH did the methodology and writing-reviewing and editing. FC, BZ, SS, JW, GZ, and ZY did the data collection. FC, BZ, SS, GZ, JW, and WH did the formal analysis. FC and WH wrote-original draft preparation. ZN and WH did the supervision. All authors have read and agreed to the published version of the manuscript.

## Conflict of Interest

The authors declare that the research was conducted in the absence of any commercial or financial relationships that could be construed as a potential conflict of interest.
